# Obesity and Circulating Levels of Vitamin D before and after Weight Loss Induced by a Very Low-Calorie Ketogenic Diet

**DOI:** 10.3390/nu13061829

**Published:** 2021-05-27

**Authors:** Silvio Buscemi, Carola Buscemi, Davide Corleo, Giovanni De Pergola, Rosalia Caldarella, Francesco Meli, Cristiana Randazzo, Salvatore Milazzo, Anna Maria Barile, Giuseppe Rosafio, Valentina Settipani, Sabina Gurrera, Antonio Maria Borzì, Marcello Ciaccio

**Affiliations:** 1 Dipartimento di Promozione della Salute, Materno-Infantile, Medicina Interna e Specialistica di Eccellenza (PROMISE), University of Palermo, 90133 Palermo, Italy; buscemi.carola@gmail.com (C.B.); davidecorleo@gmail.com (D.C.); rosalia.caldarella@policlinico.pa.it (R.C.); francesco.meli@unipa.it (F.M.); randazzocristiana@yahoo.it (C.R.); samila1918@gmail.com (S.M.); annamaria.barile@unipa.it (A.M.B.); giuseppe.rosafio@unipa.it (G.R.); valentina.settipani@unipa.it (V.S.); sabinagurrera08@gmail.com (S.G.); antoniomaria.borzi@unipa.it (A.M.B.); 2 Unit of Clinical Nutrition, Policlinico University Hospital, 90127 Palermo, Italy; 3 Department of Biomedical Science and Human Oncology, School of Medicine, Policlinico, University of Bari, 70124 Bari, Italy; gdepergola@libero.it; 4 Unit of Laboratory Medicine, AOU Policlinico “P. Giaccone”, 90127 Palermo, Italy; marcello.ciaccio@unipa.it; 5 Dipartimento di Biomedicina, Neuroscienze e Diagnostica Avanzata (BIND), University of Palermo, 90133 Palermo, Italy

**Keywords:** ketogenic diet, vitamin D, obesity, parathormone, fat mass

## Abstract

**Background:** Vitamin D plays a pivotal role in calcium and phosphorus metabolism, also influencing bone tissue. Several studies have reported that vitamin D blood levels were significantly lower in people with obesity, probably due to its uptake by the adipose tissue. Clinical studies that investigated the changes of circulating levels of vitamin D following weight loss reported controversial data. A very low-calorie ketogenic diet is acknowledged as a reliable treatment to achieve a rapid weight loss. Therefore, we investigated the effect of weight loss, consequent to a very low-calorie ketogenic diet, on vitamin D blood concentrations. **Methods:** A cohort of 31 people with obesity underwent a very low-calorie ketogenic diet for 10–12 weeks. The serum concentrations of vitamin D, parathormone, calcium and phosphorous were measured before and after weight loss; they were compared to a control group of 20 non-obese, non-diabetic, age- and gender-matched persons. **Results:** Patients with obesity had a higher habitual intake of vitamin D than the control group (*p* < 0.05). However, the vitamin D blood levels of the obese group were significantly lower than those of the control group (*p* < 0.005) and they increased after weight loss (*p* < 0.001). At baseline, vitamin D blood concentrations of the persons with obesity were significantly correlated with both fat mass–kg (r = −0.40; *p* < 0.05) and body mass index (r = −0.47; *p* < 0.01). Following very low-calorie ketogenic diet, the change in vitamin D serum concentrations was correlated only with the change in fat mass–kg (r = −0.43; *p* < 0.01). **Conclusion:** This study confirmed that patients with obesity have lower vitamin D levels that normalize after significant weight loss, supporting the hypothesis that vitamin D is stored in the adipose tissue and released following weight loss.

## 1. Introduction

Vitamin D is a fat-soluble vitamin that is essential for the metabolism of calcium and phosphorus, and the regulation of bone tissue [1]. Additionally, vitamin D has been acknowledged to influence different functions with regard to the immune, nervous, cardiovascular and endocrine systems [2,3]. Despite different foods contain vitamin D (i.e., fish), about 80% of the daily requirement of this vitamin is assured by its synthesis at the skin level through sun exposure (ultraviolet B (UVB) radiation). The nutritional status of vitamin D is assessed with the serum determination of 25-hydroxy vitamin D (25(OH)D), the intermediate metabolite of calcitriol (1, 25-hydroxy vitamin D), the biologically active form of vitamin D. Additionally, the serum concentrations of parathormone (PTH) may be used as an indirect measure of vitamin D status. The prevalence of vitamin D deficiency is very high worldwide, and, in most cases, it is attributable to reduced exposure to sunlight, impaired gut absorption or inadequate food intake [4]. In particular, low 25(OH)D levels have been extensively reported in obesity, with a prevalence ranging 40–80% [5,6], it also being inversely correlated with body weight, body mass index (BMI) and fat mass (FM) [7,8]. Given the pandemic of obesity, it is important to understand the mechanisms linking excess adiposity to low vitamin D status in order to establish effective interventions to maintain adequate 25(OH)D levels; however, the underlying mechanisms for this association have not been elucidated. As vitamin D is fat-soluble, it has been proposed that the lower levels observed in people with obesity are probably due to its uptake by the adipose tissue and the consequent clearance from plasma [9]. Moreover, it has been recently hypothesized that hepatic 25-hydroxylase could be downregulated in overweight/obesity [10]. Therefore, weight/fat loss obtained by dietary treatment is expected to increase serum vitamin D in the patients with obesity. However, few non-supplementing clinical trials measuring serum 25(OH)D concentrations in people with obesity following weight loss have been performed, and the results are conflicting [11,12]. An important limitation to organizing these studies is the difficulty of obtaining a significant weight loss in a reasonably short time in order to also avoid potential bias due to seasonal differences in sun exposure. A very low-calorie ketogenic diet (VLCKD) has been proven to be an effective and safe approach to obtaining consistent and rapid weight loss [13]. We therefore investigated whether significant weight loss obtained using VLCKD is associated with an increase in serum 25(OH)D concentrations in people with obesity.

## 2. Materials and Methods

### 2.1. Participants

From 2019 to 2020, patients with obesity were consecutively recruited to participate in an intervention study that investigated the effects of VLCKD (ISRCTN28161621; http://www.isrctn.com/ISRCTN28161621) among those attending the outpatient clinic of the unit of Clinical Nutrition, Policlinico University Hospital of the Department of Promozione della Salute, Materno-Infantile, Medicina Interna e Specialistica di Eccellenza (PROMISE; University of Palermo, Italy). Inclusion criteria were age 18–65 years, BMI (body weight (kg)/height^2^ (m^2^)) 27–39.9 kg/m^2^, and non-diabetic (glycated hemoglobin (HbA1c) < 6.5%, fasting plasma glucose (FPG) < 126 mg/dL) or type 2 diabetic people from <6 years with HbA1c ≤ 10%. Exclusion criteria were chronic ischemic heart disease; heart failure class III-IV New York Heart Association (NYHA); respiratory failure for chronic obstructive pulmonary disease, pulmonary fibrosis, bronchial asthma; renal failure stage ≥ G3a according to National Kidney Foundation (estimated glomerular filtration rate according to the Chronic Kidney Disease Epidemiology Collaboration equations < 60 mL/min/1.73 m^2^); liver cirrhosis; type 1 diabetes; history of malignant tumors in the last 10 years; presence of eating behavior disorders; alcohol abuse (>20 g/day for women; >30 g/day for men); severe psychiatric disorders/use of antipsychotic drugs; treatment with insulin, dipeptidyl peptidase-IV-inhibitors, glucagon-like peptide-1 receptor analogues, sodium-glucose transport protein 2-inhibitors or sulfonylureas; pregnancy or intention of pregnancy. Patients were compared to the participants in the ABCD2 study (ISRCTN15840340; http://www.isrctn.com/ISRCTN15840340) as controls. All participants were examined at baseline and after 10–12 weeks of dietary treatment.

The institutional Ethics Committee (“Palermo 1” of the Policlinico “P. Giaccone” University Hospital) approved the study protocol (15 October 2018, ref: # 09/2018). The study was performed in accordance with the Helsinki Declaration. All participants signed an approved informed consent.

### 2.2. Diet Intervention αnd Habitual Intake οf Vitamin D

The VLCKD in the first phase (20 ± 3 days) consisted of industrial meal replacements (Therascience Lignaform, Milano, Italy) with known protein, lipid and low carbohydrate content, and a total energy intake of 600–800 kcal/day, of which the maximum expected carbohydrate intake is 200 kcal per day (<50 g). Subsequently, conventional meals were progressively introduced, maintaining the same nutritional intake, according to a protocol described elsewhere (http://www.isrctn.com/ISRCTN28161621). In particular, in phase 2 (40 ± 5 days), 2 portions of vegetables and 1 portion of protein foods (meat, fish, eggs, cured meats) were added; in phase 3 (15 ± 3 days), 1–2 portions (125 g) of fruit were added; in phase 4 (15 ± 3 days), 1 portion of protein foods, 2 portions of dairy products, 1 portion of vegetables and 1–2 portions of fruit were added. No vitamin D supplementation was administered. All participants were advised to engage in regular physical activity, but no specific activity information was provided.

The habitual intake of vitamin D (µg/day) in the last year was assessed with a previously validated local population medium-length Food Frequency Questionnaire (FFQ) as reported elsewhere [14].

### 2.3. Anthropometric αnd Clinical Measurements

Anthropometric measurements were performed after overnight fasting at 8:00 a.m. with participants lightly dressed. After measurement of height and body weight (SECA Gmbh, Hamburg, Germany), BMI was calculated. Body circumferences were measured at the umbilicus (waist circumference) and at the most prominent buttock level (hip circumference); the ratio (waist-to-hip ratio (WHR)) was used as an indirect index of body fat distribution. Abdominal visceral (rectis-aorta thickness, RA; the distance between the linea alba and the anterior wall of the abdominal aorta) and subcutaneous adipose (cutis-rectis thickness, CR; the distance between the cutis and the conjunction of rectus muscles at the linea alba) sizes were also measured by means of high-resolution B-mode ultrasound (Epic 5, Philips, Cambridge, MA, USA) as described elsewhere [15]. The RA to CR ratio (RA/CR) was also considered as an indirect measure of body fat distribution. Body composition in terms of fat mass (FM) and fat-free mass (FFM) was estimated using bioelectrical impedance analysis (BIA-101 Anniversary, Akern, Florence, Italy) following the manufacturer’s equations, as previously described [16]. Two measurements of blood pressure and heart rate at 5 min intervals in a seated position were obtained (Omron M6, Omron Healthcare Co, Matsusaka, Mie, Japan).

### 2.4. Laboratory Analysis

A venous blood sample was drawn in the morning in postabsorptive fasting conditions at baseline and at the end of the study. Blood concentrations of 25(OH)D (Elecsys Vitamin D total II, Cobas; intra-assay CV = 8%; Roche Diagnostics GmbH, Mannheim, Germany), PTH (Elecsys PTH, Cobas; Roche Diagnostics GmbH, Mannheim, Germany), insulin (Elecsys insulina; Roche Diagnostics GmbH, Mannheim, Germany), FPG, HbA1c, total cholesterol (TC), high-density lipoprotein cholesterol (HDL-C), triglycerides (Tg), calcium, phosphorus and uric acid were measured using standard clinical chemistry methods (Roche Diagnostics GmbH, Mannheim, Germany). Low-density lipoprotein cholesterol (LDL-C) serum levels were calculated by the Friedewald’s equation [17]. The insulin resistance was estimated according to the homeostasis model assessment of insulin resistance (HOMA-IR) formula: fasting plasma insulin (μUI/mL), fasting plasma glucose (mmol/L)/22.5.

### 2.5. Statistical Analysis

A power analysis for the paired t-test showed that a total sample size of 15 subjects was required to detect a mean 25(OH)D serum concentration difference of 2.4 ng/mL (expected SD of difference of 3) for an average weight loss of 10 kg [11], with an α error of 0.05 and a power of 0.80. Data are presented as mean ± standard deviation for continuous variables, and as percentages for categorical variables. Student’s paired and unpaired t-test were applied when appropriate. Pearson’s r correlation coefficients were calculated to explore the associations among variables. A two-tailed *p* value < 0.05 was considered significant. All analyses were performed with Systat (Windows version 13.0; San Jose, CA, USA).

## 3. Results

**Figure 1:**
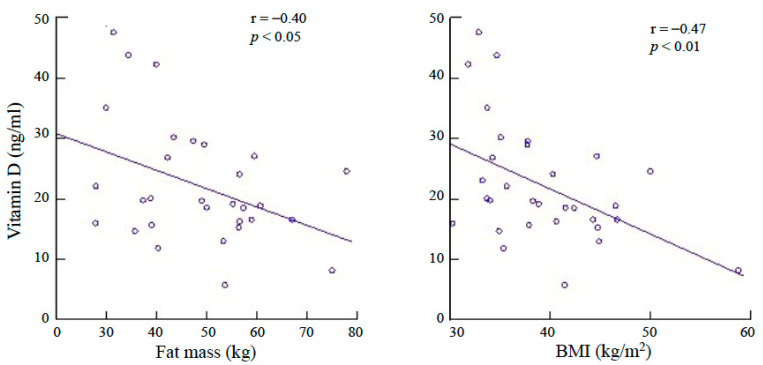
Correlations between the serum concentrations of 25-hydroxy vitamin D and fat mass (FM) and body mass index (BMI) in people with obesity.

**Figure 2:**
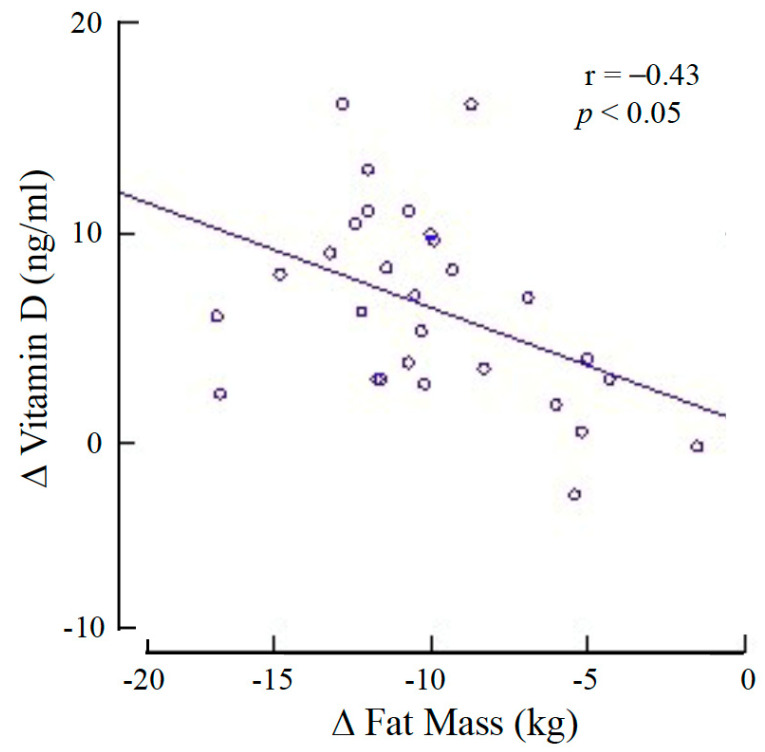
Correlation between the change (Δ) in serum concentrations of 25-hydroxy vitamin D and that of fat mass in people with obesity following very low-calorie ketogenic diet.

A total of 37 patients with obesity (12 males and 25 females) were initially selected. Among these, five (four males and one female) participants were excluded due to drop-out, and one because of treatment failure (weight loss < 5%). Finally, a total of 31 participants were evaluated and compared to a control group of 20 non-obese, non-diabetic, age-matched persons. Type 2 diabetes was diagnosed in seven patients of the obese group, whereas hypertension was diagnosed in three patients with obesity. The physical and clinical characteristics of the participants are presented in Table 1. The habitual intake of vitamin D was significantly higher (*p* < 0.05) in the patients with obesity than in the control group. The serum concentrations of 25(OH)D of the obese group were significantly lower than those of the control group (*p* < 0.005) and increased significantly after weight loss (*p* < 0.001). Before the VLCKD treatment, the serum concentrations of 25(OH)D of the persons with obesity were significantly correlated with both FM–kg (r = −0.40; *p* < 0.05) and BMI (r = −0.47; *p* < 0.01) but not with body weight, body circumferences, WHR, RA, CR, serum insulin concentrations or HOMA-IR (*p* = not significant) (Figure 1). Following VLCKD, the change in serum concentrations of 25(OH)D was correlated solely with the change in FM–kg (r = −0.43; *p* < 0.01) (Figure 2). In particular, no correlation was observed between the change in serum concentrations of 25(OH)D and the HOMA-IR value before (r = 0.13; *p* = not significant) and after (r = −0.12; *p* = not significant) weight loss. Data concerning vitamin D, biochemical and hormonal characteristics of the groups are reported on Table 2.

## 4. Discussion

The present study confirms that people with obesity have lower blood concentrations of 25(OH)D and higher concentrations of PTH than non-obese persons, despite a higher habitual intake of vitamin D. A possible explanation is that people with obesity may usually have lower skin UV sun exposure, as it has been reported that they have reduced outdoor activity during the summer and that they generally prefer to cover as much of the body with clothes as possible [18,19]. However, we found that serum concentrations of 25(OH)D were inversely correlated with measures of general adiposity as the BMI and the FM size suggesting that adipose tissue is an important influencing factor. In fact, the significant increase in 25(OH)D concentrations observed following VLCKD and the consequent weight/fat loss is in agreement with the hypothesis of our study that the low 25(OH)D concentrations observed in people with obesity are due to its uptake and storage by adipose tissue being released following fat mass reduction. In particular, the change in 25(OH)D blood concentrations was correlated solely with the change in FM, indicating the prominent role of FM as a possible depot. This might be an interesting mechanism, suggesting that adipose tissue serves as an endogenous source of vitamin D, for instance during the winter, when cutaneous production is low. A meta-regression analysis included 18 weight loss studies, 5 of which reported no change, and 1 study even reported a reduction in 25(OH)D concentrations following weight loss [11]; overall, the results demonstrated an average increase in 25(OH)D concentrations of 6 nmol/L for an average weight loss of 10 kg that was close to significance (*p* = 0.06). Our results are in agreement with these data since we found that 25(OH)D concentrations increased by an average of 4.2 ng/mL (10.5 nmol/L) for a weight loss of 15.1 kg. Our data are also in agreement with data observed in patients with obesity who underwent weight loss surgery. In fact, it is generally reported that serum concentrations of 25(OH)D increase soon after surgery [20,21] and decline in the longer term, probably due to malabsorption [22]. Intriguingly, another possible proposed mechanism leading to lower 25(OH)D in people with obesity could be the decreased expression of hepatic 25-hydroxylase, as observed by Roizen et al. in obese mice [10]; however, this hypothesis has not been assessed in humans yet. We could not find any significant correlation between 25(OH)D concentrations and different measures of visceral and subcutaneous fat, thus suggesting that it is the total FM size rather than body fat distribution that influences the circulating levels of 25(OH)D.

To date, there is no evidence that VLCKD has a specific effect on 25(OH)D concentrations as compared with other dietary approaches. To the best of our knowledge, only one study has investigated the change in 25(OH)D concentrations in people with obesity before and after weight loss using VLCKD [23]; even in that case, a significant increase in 25(OH)D concentrations was found, despite measurements being performed 1 year later. 

As expected, we also observed that people with obesity exhibited higher PTH blood concentrations parallel to lower levels of 25(OH)D [24] in order to maintain a normal calcium–phosphorus product. Therefore, our study seems to suggest that the PTH concentrations could, at least in part, regulate calcium concentrations as a consequence of 25(OH)D deficiency, since they significantly decreased following body weight loss. Since PTH induces osteoclasts activity, this may well represent a further mechanism contributing to osteoporosis in people with obesity [25].

This study has some limitations. We did not investigate the mechanisms that control the release of 25(OH)D from the adipose tissue and that may involve vitamin D receptors, enzymatic and genetic factors [26], and we did not measure the sub-fractions of vitamin D either. 

On the other hand, this study has the merit of having investigated a still unclarified topic related to significant weight/fat loss in a very short time, thus avoiding possible confounding factors associated with seasonal changes in UV exposure and dietary intake.

## 5. Conclusions

Low 25(OH)D levels, balanced by increased PTH concentrations, characterize people with obesity. Significant weight loss with a reduction in FM normalizes both serum 25(OH)D and PTH blood levels. These results seem to indicate that vitamin D is captured and stored in the adipose tissue of patients with obesity and released following weight loss. Additional studies are needed to clarify the mechanisms that regulate the kinetic of vitamin D in the adipose tissue of persons with obesity. Additionally, further studies are warranted to investigate if these aspects seen in people with obesity are of clinical relevance and need pharmacologic correction.

## Figures and Tables

**Table 1:** Physical and clinical characteristics of patients with obesity before and after dietary treatment.

	Control Group	*p* ^a^	VLCKD (*n* = 31)		*p* ^b^
	(*n* = 20)		Before	After	
Age (years)	46 ± 8	0.29	43 ± 11		
Males (%)	25.0		25.6		
Body weight (kg)	71.2 ± 14.8	<0.001	104.3 ± 16.8	89.2 ± 15.5	<0.001
BMI (kg/m^2^)	24.1 ± 2.9	<0.001	39.4 ± 6.3	33.7 ± 5.9	<0.001
Circumferences					
waist (cm)	93.0 ± 10.1	<0.001	119.5 ± 11.9	107.1 ± 13.2	<0.001
hip (cm)	104.3 ± 7.7	<0.001	124.8 ± 13.0	115.8 ± 12.0	<0.001
WHR	0.89 ± 0.05	<0.001	0.96 ± 0.08	0.93 ± 0.10	<0.005
BIA					
fat mass (%)	29.6 ± 5.7	<0.001	45.8 ± 7.8	41.3 ± 8.3	<0.001
fat-free mass (kg)	49.8 ± 9.7	<0.05	56.2 ± 8.6	52.4 ± 9.3	<0.001
phase angle (°)	6.8 ± 0.7	0.62	6.9 ± 0.7	6.8 ± 0.8	0.20
Ultrasound thickness					
cutis-rectis (cm)	3.2 ± 0.6	<0.001	4.5 ± 1.2	4.2 ± 1.2	<0.001
rectis-aorta (cm)	4.2 ± 1.7	<0.001	8.9 ± 3.6	5.6 ± 2.7	<0.001
Blood pressure					
systolic (mmHg)	119 ± 16	0.55	122 ± 18	115 ± 10	<0.05
diastolic (mmHg)	81 ± 10	0.19	85 ± 11	78 ± 8	<0.001
Heart rate (beats/min)	70 ± 10	<0.005	81 ± 12	78 ± 11	0.18

Mean ± SD; ^a^ Student’s unpaired t-test vs. patients with obesity before VLCKD; ^b^ Student’s paired *t*-test. BIA, bioimpedance analysis; BMI: body mass index; VLCKD: very low-calorie ketogenic diet; WHR: waist-to-hip ratio.

**Table 2:** Fasting serum concentration of the hormonal and biochemical variables of patients with obesity before and after dietary treatment.

	Control Group	*p* ^a^	VLCKD (*n* = 31)		*p* ^b^
	(*n* = 20)		Before	After	
Habitual intake of vitamin D (µg/day)	1.12 ± 0.83	<0.05	1.61 ± 0.56		
Serum concentration of:					
HbA_1_c (%)	5.5 ± 0.4	0.07	6.1 ± 1.4	5.7 ± 0.5	<0.05
glucose (mg/dL)	89 ± 10	0.09	108 ± 48	95 ± 17	0.07
insulin (μUI/mL)	9.1 ± 4.4	0.15	12.6 ± 10	7.9 ± 5.6	<0.005
HOMA-IR	1.92 ± 0.46	<0.001	3.76 ± 0.94	1.74 ± 0.68	<0.001
cholesterol (mg/dL)	202 ± 51	0.05	178 ± 36	151 ± 25	<0.001
HDL-C (mg/dL)	67 ± 20	<0.05	55 ± 13	46 ± 13	<0.05
triglycerides (mg/dL)	96 ± 51	0.32	113 ± 64	84 ± 35	<0.001
25-hydroxy vitamin D (ng/mL)	29.7 ± 6.7	<0.005	21.6 ± 9.9	25.8 ± 10.4	<0.001
PTH (pg/mL)	28.5 ± 8.2	<0.001	41.0 ± 11.7	32.0 ± 13.6	<0.01
calcium (mg/dL)	9.1 ± 0.3	<0.01	9.4 ± 0.4	9.5 ± 0.4	0.06
phosphorus (mg/dL)	3.6 ± 0.5	0.17	3.4 ± 0.5	3.6 ± 0.4	<0.05

Mean ± SD. ^a^ Student’s unpaired t-test vs. patients with obesity before VLCKD. ^b^ Student’s paired *t*-test. HbA_1_c: glycated hemoglobin; HDL-C: high-density lipoprotein cholesterol; HOMA-IR: homeostasis model assessment of insulin resistance; PTH: parathormone; VLCKD: very low-calorie ketogenic diet.

## Data Availability

All data regarding this study are available upon appropriate request to the corresponding author by the e-mail: silvio.buscemi@unipa.it.
